# Increased Otoferlin Expression in B Cells Is Associated with Muscle Weakness in Untreated Juvenile Dermatomyositis: A Pilot Study

**DOI:** 10.3390/ijms241310553

**Published:** 2023-06-23

**Authors:** Ameera Bukhari, Amer Khojah, Wilfredo Marin, Andrey Khramtsov, Galina Khramtsova, Christopher Costin, Gabrielle Morgan, Prathyaya Ramesh, Marisa S. Klein-Gitelman, I. Caroline Le Poole, Lauren M. Pachman

**Affiliations:** 1 College of Science, Taif University, Taif 21944, Saudi Arabia; 2 Department of Pediatrics, College of Medicine, Umm Al-Qura University, Makkah 24381, Saudi Arabia; 3 Division of Pediatric Rheumatology, Ann & Robert H. Lurie Children’s Hospital of Chicago, Chicago, IL 60611, USA; 4 Stanley Manne Children’s Research Institute, Ann & Robert H. Lurie Children’s Hospital of Chicago, Chicago, IL 60611, USAgkhramtsova@luriechildrens.org (G.K.); 5 Department of Pathology, Ann & Robert H. Lurie Children’s Hospital of Chicago, Chicago, IL 60611, USA; 6 Feinberg School of Medicine, Northwestern University, Chicago, IL 60611, USA; 7 Department of Dermatology, Microbiology & Immunology, Northwestern University, Chicago, IL 60611, USA

**Keywords:** B cell, otoferlin, juvenile dermatomyositis, disease activity scores, muscle weakness

## Abstract

Otoferlin mRNA expression is increased in JDM patients’ PBMCs and muscle compared to healthy controls. This study aims to evaluate the role of otoferlin in JDM disease pathophysiology and its association with disease activity in untreated children with JDM. A total of 26 untreated JDM (88.5% female, 92.3% white, non-Hispanic) and 15 healthy controls were included in this study. Otoferlin mRNA expression was determined by qRT-PCR before and a few months after therapy. Detailed flow cytometry of various cell surface markers and cytoplasmic otoferlin was performed to identify cells expressing otoferlin. In addition, muscle otoferlin expression was evaluated in situ in six untreated JDM patients and three healthy controls. There was a significant increase in otoferlin expression in JDM children compared to controls (Median 67.5 vs. 2.1; *p* = 0.001). There was a positive correlation between mRNA otoferlin expression and the following disease activity markers: disease activity scores (DAS)-total (r_s_ = 0.62, *p* < 0.001); childhood myositis assessment scale (CMAS) (r_s_ = −0.61, *p* = 0.002); neopterin (r_s_ = 0.57, *p* = 0.004) and von Willebrand factor antigen (vWF: Ag) (r_s_ = 0.60, *p* = 0.004). Most of the otoferlin-positive cells were unswitched B cells (63–99.4%), with 65–75% of them expressing plasmablast markers (CD19^+^, IgM^+^, CD38^hi^, CD24^−^). The findings of this pilot study suggest that otoferlin expression is associated with muscle weakness, making it a possible biomarker of disease activity. Additionally, B cells and plasmablasts were the primary cells expressing otoferlin.

## 1. Introduction

Juvenile dermatomyositis (JDM) is a rare pediatric inflammatory myopathy characterized by muscle weakness, skin rash, and microvascular injury [1]. The estimated incidence of JDM in the USA is 2.7–3.4 cases per million annually [2]. The underlying mechanisms of JDM are complex and involve both the adaptive and innate immune systems [1]. For example, most untreated JDM patients have elevated neopterin levels, a marker for macrophage activation and interferon γ production [3]. Muscle biopsies of children with JDM frequently show infiltration of lymphocytes, macrophages, and plasmacytoid dendritic cells [1,4,5]. Additionally, there is evidence indicating that inherited B cell defects increase the risk of JDM [6], and more than 50% of children with JDM exhibit myositis-specific antibodies (MSA), which are linked to distinct disease phenotypes [7]. Furthermore, mitochondria dysfunction and neutrophil extracellular trap (NET) formation has been implicated in the disease pathophysiology, especially in JDM patients with calcinosis [8,9,10]. The exact cause of the inflammatory response is unclear but likely due to a combination of genetic susceptibility and appropriate environmental triggers [11]. To better understand the pathophysiology of JDM, our group performed RNA-Seq from peripheral blood mononuclear cells (PBMCs) of active JDM before treatment and age-matched controls [12]. The RNA-Seq data showed a marked increase in the expression of type 1 interferon-responsive genes, which is consistent with the other studies [12,13]. Another significantly upregulated gene in the untreated JDM PBMCs compared to control was otoferlin (OTOF; 23.6 FC) [12]. Otoferlin is a member of the ferlin family [14] and is essential to regulating calcium-sensitive exocytosis in inner ears’ sensory hair cells [15]. Defective otoferlin leads to profound hearing impairment in humans [16]. Interestingly, mutations in dysferlin, another member of the ferlin family, lead to limb–girdle muscular dystrophy type 2B and Miyoshi myopathy by altering calcium homeostasis in the skeletal muscle [17,18]. Although the role of otoferlin in muscle inflammation is not yet known, it is suspected to be calcium-flux-related. A previous RNA-Seq study demonstrated significant increases in otoferlin expression in PBMCs from untreated children with JDM compared to matched controls [12].

This study aims to further understand the role of otoferlin in JDM pathophysiology by identifying the cells that express otoferlin and examining its association with disease activity in untreated children with JDM.

## 2. Results

**Figure 1:**
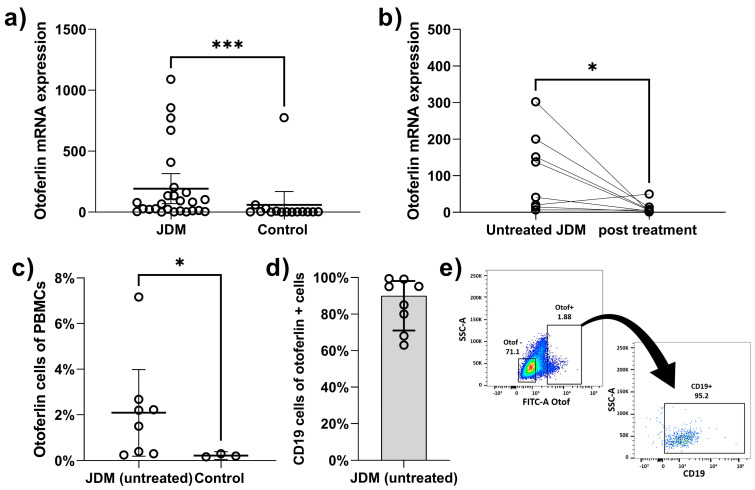
Otoferlin expression in PBMCs from JDM children. (**a**) There was a significant increase in otoferlin expression in JDM children compared to controls (median 73.2 vs. 2.1; *p* = 0.001). (**b**) The otoferlin expression decreased significantly after treatment (paired *t*-test; mean 109.3 vs. 11.3, *p* = 0.04). (**c**) Increased percentage of otoferlin-positive lymphocytes in JDM (median 1.9% vs. 0.2% *p* = 0.03). (**d**) The majority of the otoferlin-positive cells were B cells (63–99.4%). (**e**) Example of plot chart showing the percentage of CD19 + ve cells out of otoferlin positive cells. *** means *p* < 0.001 and * means *p* < 0.05.

**Figure 2:**
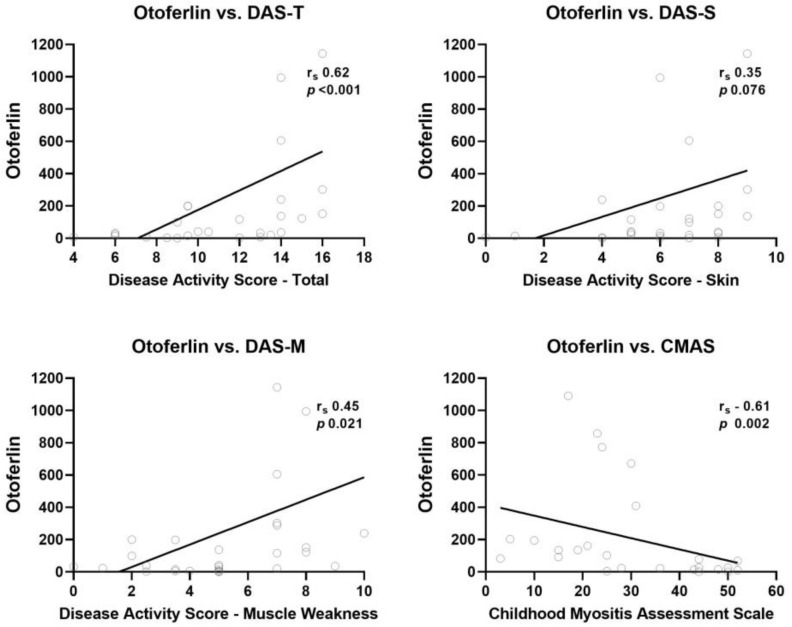
Otoferlin expression and clinical disease activity indicators. There was a positive correlation between otoferlin expression and clinical disease activity indicators: DAS-total (r_s_ = 0.62, *p* < 0.001); DAS-skin (r_s_ = 0.35, *p* = 0.076); DAS-muscle weakness (r_s_ = 0.45, *p* = 0.021); and CMAS (r_s_ = −0.61, *p* = 0.002).

There was a significant increase in the otoferlin expression in JDM children compared to controls (median 73.2 vs. 2.1; *p* = 0.001) (Figure 1a). The otoferlin expression decreased significantly after 2–3 months of treatment (Figure 1b). There was no significant correlation between mRNA otoferlin expression and age or duration of untreated disease (*p* = 0.956 and *p* = 0.229, respectively) in JDM. Furthermore, there was no significant association between the MSAs and otoferlin expression (Supplemental Figure S4). However, higher otoferlin expression was associated with increased disease activity (Table 1). For example, there was a positive correlation between otoferlin expression and clinical disease activity indicators, such as DAS-total (r_s_ = 0.62, *p* < 0.001), DAS-muscle weakness (r_s_ = 0.452, *p* = 0.021), and CMAS (r_s_ = −0.611, *p* = 0.002) (Figure 2). Because of the strong correlation between otoferlin expression and muscle weakness (assessed by DAS -muscle weakness and CMAS), we evaluated the association between otoferlin expression and muscle enzyme levels. Higher otoferlin expression was associated with increased serum lactate dehydrogenase (LDH) and aldolase but not creatine phosphokinase (CK) or aspartate aminotransferase (AST) (Table 1). 

We evaluated the relationship between inflammatory markers, such as von Willebrand factor antigen (vWF: Ag), erythrocyte sedimentation rate (ESR), neopterin, and otoferlin expression. vWF: Ag level and serum neopterin positively correlated with otoferlin expression (r_s_ = 0.602, *p* = 0.004, and r_s_ = 0.57, *p* = 0.004, respectively). Because of the positive correlation between otoferlin and serum neopterin, a marker of macrophage activation upon interferon γ stimulation, we evaluated the relationship between otoferlin expression and serum CXCL10, also known as interferon γ-induced protein 10 (IP-10), in 7 JDM subjects. There was a positive correlation between CXCL10 and otoferlin expression (r_s_ = 0.8, *p* = 0.03) (Supplemental Figure S5). Of note, there was no significant correlation between ESR level and otoferlin expression. This is not completely surprising as the median ESR of the study subjects was 10 mm/h, which is within the normal range (0–20 mm/h).

To investigate which cell was expressing otoferlin, we first evaluated the correlation between the otoferlin expression and various lymphocyte subsets. There was a negative correlation between the percentage of NK cells and otoferlin expression (r_s_ = −0.439, *p* = 0.025). Then, we performed flow cytometry with cytoplasmic otoferlin staining in eight JDM patients and three controls. Consistent with the RNA expression data, JDM patients had a significantly higher percentage of otoferlin-positive cells (median 1.9% vs. 0.2% *p* = 0.03) (Figure 1c). Most of the otoferlin-positive cells were positive for CD19 staining, suggesting that they were B cells (Figure 1d,e). Detailed B cell phenotyping in two samples showed that these B cells were IgD^+^, IgM^+^ CD27^−^ naive B cells, with 65–75% of them expressing plasma blast markers (CD19^+^, IgM^+^, CD38^hi^, CD24^−^). 

**Figure 3:**
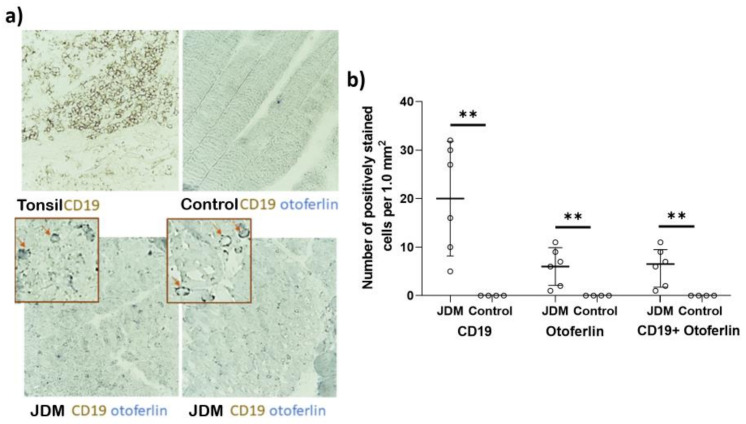
Immunohistochemical staining otoferlin and CD19 staining of muscle tissues. (**a**) Example of otoferlin and CD19 staining of muscle tissues from 2 JDM subjects and a control sample. Tonsillar tissue was included as a positive control for CD19 and a negative control for otoferlin expression. Of note, magnification level is ×200. (**b**) Here is a significant increase in CD19 +ve otoferlin +ve cells in the JDM subjects compared to the control. ** means *p* < 0.01.

We performed a tissue staining study on the muscle biopsy of six untreated JDM patients and three controls to assess the infiltration of otoferlin-positive B cells in the muscle. Our results showed that the mean number of CD19 + ve cells (B cells) per mm^2^ was 20 cells/mm^2^ in JDM patients, which was not present in the control group. Additionally, the mean number of otoferlin + ve cells in JDM was six cells/mm^2^ and was absent in the control group. All of the otoferlin + ve cells were co-stained for CD19, indicating that they were B cells. The number of CD19 + ve and otoferlin + ve cells was significantly higher in JDM patients compared to the control group (*p* = 0.013), as determined by an unpaired *t*-test with Welch’s correction (Figure 3).

## 3. Discussion

This study documented the presence of otoferlin-positive cells in the peripheral blood of JDM, which are not typically found in healthy controls. Although the exact role of these cells in the pathophysiology of JDM is not completely clear, we showed a positive correlation between otoferlin expression and various disease activity indicators, especially in relation to muscle weakness (Table 1). Furthermore, muscle biopsies of JDM patients show an increased number of otoferlin-positive cells compared to controls, which suggests the potentially important role of these cells in muscle inflammation. Most of the otoferlin-positive cells were B cells, identified as IgD^+^, IgM^+^ CD27^−^ naive B cells, with more than 50% expressing plasmablast markers (CD19^+^, IgM^+^, CD38^hi^, and CD24^−^).

Otoferlin is a member of the ferlin family, which are large proteins involved in Ca sensing and vesicular trafficking [19,20]. Other members of this family include dysferlin and myoferlin, among others [14,21]. Otoferlin is essential in regulating calcium-sensitive exocytosis in the inner ears’ sensory hair cells [15]. Defects in the otoferlin function can lead to profound hearing loss in humans [16,22]. The role of otoferlin in inflammation and B cell biology has not been previously identified but was suspected to be based on a previous RNA-seq study that showed significant increases in otoferlin expression in PBMCs compared to controls [12]. This unbiased approach led to the recognition of the potential role of otoferlin in muscle inflammation in JDM, which was explored in this manuscript. The correlation between otoferlin expression and disease activity indicators suggests its potential use as a biomarker for disease activity in JDM, particularly for muscle weakness. Biomarkers are important to optimize treatment and reduce long-term glucocorticoid side effects, such as obesity and decreased muscle mass [11,23]. Additionally, there is a strong correlation between otoferlin expression and vWF: Ag level, a marker for severe JDM that can indicate vascular injury [24,25]. Finally, otoferlin expression has an inverse correlation with the peripheral NK cell count. Low NK cell count has been associated with an increase in disease activity of the orbital myositis [26]. In addition, a recent study has shown that children with JDM have lower than normal NK cell counts, particularly after the onset of the COVID-19 pandemic [27]. Overall, these findings expand upon existing knowledge of otoferlin and its potential role as a biomarker in JDM.

The identification of B cells and plasmablasts as the primary cells expressing otoferlin is also an important finding of this study. B cells are known to play an important role in the pathophysiology of JDM, as evidenced by the influence of autoantibodies on the disease phenotype [1,28] and B cell infiltration of muscle tissue in active disease, which is also confirmed in this study. Furthermore, B cell-depleting agent [29,30,31] and intravenous immunoglobulin (IVIG) [32,33] have been shown to be effective therapies when first-line therapy fails. Expanded naïve B cells with transitional markers (CD19^+^CD24^hi^CD38^hi^) have been observed in untreated JDM patients and correlated with type I interferon signatures [34]. These cells are activated through the toll-like receptor 7 (TLR7) and interferon α [34]. The otoferlin expression correlated to the levels of serum neopterin and CXCL10, a protein known to be induced by interferon, highlighting the significance of interferon in B cell dysregulation. This is consistent with previous research, indicating the crucial role of type 1 interferon signaling in the development of autoreactive B cells [34,35]. Although the presence of otoferlin-positive B cells in the peripheral blood and muscle tissue of JDM patients suggests its possible role in the disease process, further research is needed to establish the exact role of otoferlin in B cell dysregulation and its connection to disease activity.

This study has several limitations that should be noted. First, it is a pilot study with a small sample size, which could limit the generalizability of the results. Secondly, the study was not designed to detect any subtle differences between different MSA subgroups of JDM. Lastly, further functional testing and studies in knockout mouse models [36] were not conducted; therefore, the exact mechanism by which otoferlin affects B cell activation remains unclear.

## 4. Materials and Methods

### 4.1. Study Subjects

This IRB-approved study was conducted at the Ann & Robert H. Lurie Children’s Hospital of Chicago (IRB# 2008-13457). We included 26 JDM patients and 15 age-matched controls in this study. All study patients fulfilled the EULAR/ACR 2017 classification criteria for the definite JDM [37] and Bohan and Peter’s criteria [38,39]. All study participants had samples available before initiating medical therapy. Clinical variables, including age, sex, duration of untreated disease, childhood myositis assessment scale (CMAS), and disease activity scores (DAS skin, muscle weakness, total) [40] were obtained from the Ann & Robert H. Lurie Children’s Hospital of Chicago Juvenile Myositis Registry REDCap database. The demographic data of this’s study subjects are presented in Table 2. MSAs were assessed by immunoprecipitation and immunodiffusion at Oklahoma Medical Research Foundation [41]. A competitive enzyme-linked immunosorbent assay (ALPCO diagnostics kit) was used to measure the serum neopterin [3]. Fifteen age-matched healthy control volunteers were enrolled in the study (IRB# 2001-11715) after undergoing screening to confirm the absence of medical illnesses. As compensation for their participation in the study, nailfold capillaroscopy, and blood donation, they were provided with a $25 gift card.

### 4.2. Otoferlin Expression and Flow Cytometry

Otoferlin expression was determined by qRT-PCR in PBMCs from untreated children with JDM and healthy controls [42]. To identify cells expressing otoferlin, flow cytometry was done on eight children with JDM and three healthy controls. The cells were first stained with live dead dye (eBioscience—eFluor 780) to exclude dead cells. The cell viability was >90% in all the samples. The following surface markers were determined (CD45, CD3, CD19, CD16, CD56, CD14 and CD11b) to characterize the otoferlin-positive cells. We fixed and permeabilized the cells and then stained them for cytoplasmic otoferlin expression. The otoferlin-positive cells were primarily B cells; more detailed flow cytometry was performed with the following markers (CD19, IgM, IgD, IgG, CD27, CD21, CD24, and CD38) to characterize these B cells further. An example of the gating strategy of both experiments is found in the Supplemental Material (Supplemental Figures S1 and S2). 

### 4.3. Immunohistochemistry

For immunoenzymatic staining, 8 um of frozen sections of muscle tissue biopsies from untreated JDM patients and healthy female pediatric controls were air-dried and stored at −20 °C until use. Tissue sections were incubated with mouse IgG2b anti-human CD19 antibody (clone A17136C, Biolegend, San Diego, CA, USA) and mouse IgG1 anti-human otoferlin antibody (clone 13A9, Abcam, Cambridge, UK). Alkaline phosphatase staining was developed in the presence of Fast BlueBB substrate (Millipore Sigma, St. Louis, MO, USA), followed by the development of horseradish peroxidase by adding either 3,3′-diaminobenzidine tetrahydrochloride substrate (Leica Biosystems, Vista, CA, USA) or 3-Amino-9-Ethylcarbazole (AEC) detection solution (Abcam) [14,42]. The histological slides were digitized using NanoZoomer S210 Digital Slide Scanner (Hamamatsu), scanning resolution 40× mode, 0.23 µm/pixel. The total number of positively stained cells per 1.0 mm^2^ was estimated on representative histologic sections. Using QuPath v0.4.1 software, a grid composed of 0.1 mm^2^ fields of view was generated [43]. The number of positively stained cells per 10 fields (1.0 mm^2^) of view was evaluated (Supplemental Figure S3). Two observers performed quantitative analysis of the tissue specimen without knowledge of specimen identification. The tissues were evaluated for the expression of CD19 and otoferlin. Scoring was based on the count of the cells stained with CD19, otoferlin, and double staining per mm^2^. To validate the specificity of the CD19 antibody, human tonsillar tissue was used as a positive control, as it is known to express CD19. Additionally, human tonsillar tissue was employed as a negative control for otoferlin staining, as it is known not to express otoferlin. Isotypic IgG was used as a negative control for staining.

### 4.4. Statistical Analysis 

Given the non-parametric distribution of the otoferlin expression data, Spearman’s correlation was used to assess the correlation between mRNA expression and various clinical and laboratory disease activity indicators. Similarly, the Mann–Whitney test was used to compare otoferlin mRNA expression between two independent groups, such as sex. IBM SPSS Statistics and GraphPad Prism 8 software were used to conduct statistics and generate the figures.

## 5. Conclusions

The results of this pilot study suggest that otoferlin expression is increased in JDM patients compared to healthy controls and is positively correlated with disease activity markers. Furthermore, the study identified B cells and plasmablasts as the primary cells expressing otoferlin. 

## Figures and Tables

**Table 1:** Correlation of peripheral blood otoferlin mRNA expression in 29 untreated children with JDM disease activity markers and flow cytometry results.

Clinical Findings	Median (25%ile–75%ile)	Spearman’s Correlation Coefficient	*p*-Value
**Clinical disease activity indicator**			
Disease activity score (DAS) total	12 (9.1–14)	**0.620**	**<0.001**
Disease activity score skin	6.3 (5–8)	0.354	0.076
Disease activity score muscle weakness	5 (3.5–7)	**0.452**	**0.021**
Childhood myositis assessment scale (CMAS)	28 (18–44)	**−** **0.611**	**0.002**
Nailfold capillary end row loops (ERL) (#/mm)	4 (3.5–5.9)	0.128	0.532
**Laboratory disease activity indicator**			
Neopterin (nmol/L)	16.5 (12–27)	**0.570**	**0.004**
Erythrocyte sedimentation rate (ESR) (mm/h)	10 (8.5–24.5)	0.246	0.359
Von Willebrand factor antigen (vWF: Ag) (%)	143 (96–195)	**0.602**	**0.004**
**Muscle enzymes**			
Creatine phosphokinase (CK) (IU/L)	132.5 (98.5–365.3)	0.090	0.699
Aspartate aminotransferase (AST) (IU/L)	50 (39.5–64.5)	0.417	0.068
Lactate dehydrogenase (LDH) (IU/L)	324 (285.5–536)	**0.580**	**0.007**
Aldolase (U/L)	9.9 (7.5–13.7)	**0.624**	**0.006**
**Flow cytometry**			
Total T cells (CD3^+^)	63 (53–69)	−0.223	0.275
T helper cells (CD3^+^ CD4^+^)	42 (34.5–49)	0.018	0.929
T cytotoxic cells (CD3^+^ CD8^+^)	19 (16–23)	−0.214	0.293
B cells (CD19^+^)	31 (23–42)	0.370	0.063
NK cells (CD16^+^/CD56^+^)	4 (3–7.5)	**−** **0.439**	**0.025**

**Table 2:** Demographic characteristics of study subjects.

	JDM Patients (*n* = 26)	Healthy Controls (*n* = 15)	*p*-Value
Age			
<6 years old	12 (46.2%)	5 (33.3%)	0.236
6–12 years old	11 (42.3%)	5 (33.3%)	
>12 years old	3 (11.5%)	5 (33.3%)	
Sex			
Female	23 (88.5%)	8 (53.3%)	0.012
Male	3 (11.5%)	7 (46.7%)	
Race/ethnicity			
White, non-Hispanic	24 (92.3%)	10 (66.7%)	0.099
White, Hispanic	0 (0%)	3 (20 %)	
African American	1 (3.8%)	1 (6.7%)	
Others	1 (3.8 %)	1 (6.7%)	
Myositis specific antibodies			
P155/140	11 (42.3%)		
MJ	4 (15.4%)		
Mi2	4 (15.4%)		
MDA5	2 (7.7%)		
Negative	4 (15.4%)		
Not tested	1 (3.8%)		

## Data Availability

The data that support the findings of this study are available from the corresponding author upon reasonable request.

## Supplementary Materials

The supporting information can be downloaded at: https://www.mdpi.com/article/10.3390/ijms241310553/s1.

## Abbreviations

JDM, juvenile dermatomyositis; PBMCs, peripheral blood mononuclear cells; DAS, disease activity score; CMAS, childhood myositis assessment scale; MSA, myositis-specific antibodies; NET, neutrophil extracellular traps; qRT-PCR, real-time quantitative reverse transcription PCR; vWF: Ag, von Willebrand factor antigen; CXCL10, C-X-C motif chemokine ligand 10; IP-10, interferon γ-induced protein 10; NK cells, natural killer cells; CK, creatine phosphokinase; AST, aspartate aminotransferase; LDH, lactate dehydrogenase; ESR, erythrocyte sedimentation rate.

## References

[1] Pachman L.M., Khojah A.M. (2018). Advances in Juvenile Dermatomyositis: Myositis Specific Antibodies Aid in Understanding Disease Heterogeneity. J. Pediatr..

[2] Mendez E.P., Lipton R., Ramsey-Goldman R., Roettcher P., Bowyer S., Dyer A., Pachman L.M., Niams Juvenile dm Registry Physician Referral Group (2003). US incidence of juvenile dermatomyositis, 1995–1998: Results from the National Institute of Arthritis and Musculoskeletal and Skin Diseases Registry. Arthritis Rheum..

[3] Khojah A., Morgan G., Pachman L.M. (2021). Clues to Disease Activity in Juvenile Dermatomyositis: Neopterin and Other Biomarkers. Diagnostics.

[4] Sag E., Kale G., Haliloglu G., Bilginer Y., Akcoren Z., Orhan D., Gucer S., Topaloglu H., Ozen S., Talim B. (2021). Inflammatory milieu of muscle biopsies in juvenile dermatomyositis. Rheumatol. Int..

[5] Wedderburn L.R., Varsani H., Li C.K., Newton K.R., Amato A.A., Banwell B., Bove K.E., Corse A.M., Emslie-Smith A., Harding B. (2007). International consensus on a proposed score system for muscle biopsy evaluation in patients with juvenile dermatomyositis: A tool for potential use in clinical trials. Arthritis Rheum..

[6] Padem N., Wright H., Fuleihan R., Garabedian E., Suez D., Cunningham-Rundles C., Marsh R.A., Khojah A. (2022). Rheumatologic diseases in patients with inborn errors of immunity in the USIDNET registry. Clin. Rheumatol..

[7] Satoh M., Tanaka S., Ceribelli A., Calise S.J., Chan E.K. (2017). A Comprehensive Overview on Myositis-Specific Antibodies: New and Old Biomarkers in Idiopathic Inflammatory Myopathy. Clin. Rev. Allergy Immunol..

[8] Duvvuri B., Pachman L.M., Morgan G., Khojah A.M., Klein-Gitelman M., Curran M.L., Doty S., Lood C. (2020). Neutrophil Extracellular Traps in Tissue and Periphery in Juvenile Dermatomyositis. Arthritis Rheumatol..

[9] Seto N., Torres-Ruiz J.J., Carmona-Rivera C., Pinal-Fernandez I., Pak K., Purmalek M.M., Hosono Y., Fernandes-Cerqueira C., Gowda P., Arnett N. (2020). Neutrophil dysregulation is pathogenic in idiopathic inflammatory myopathies. JCI Insight.

[10] Duvvuri B., Pachman L.M., Hermanson P., Wang T., Moore R., Ding-Hwa Wang D., Long A., Morgan G.A., Doty S., Tian R. (2023). Role of mitochondria in the myopathy of juvenile dermatomyositis and implications for skeletal muscle calcinosis. J. Autoimmun..

[11] Pachman L.M., Nolan B.E., DeRanieri D., Khojah A.M. (2021). Juvenile Dermatomyositis: New Clues to Diagnosis and Therapy. Curr. Treatm. Opt. Rheumatol..

[12] Roberson E.D.O., Mesa R.A., Morgan G.A., Cao L., Marin W., Pachman L.M. (2021). Transcriptomes of peripheral blood mononuclear cells from juvenile dermatomyositis patients show elevated inflammation even when clinically inactive. bioRxiv.

[13] Kim H., Gunter-Rahman F., McGrath J.A., Lee E., de Jesus A.A., Targoff I.N., Huang Y., O’Hanlon T.P., Tsai W.L., Gadina M. (2020). Expression of interferon-regulated genes in juvenile dermatomyositis versus Mendelian autoinflammatory interferonopathies. Arthritis Res. Ther..

[14] Redpath G.M., Sophocleous R.A., Turnbull L., Whitchurch C.B., Cooper S.T. (2016). Ferlins Show Tissue-Specific Expression and Segregate as Plasma Membrane/Late Endosomal or Trans-Golgi/Recycling Ferlins. Traffic.

[15] Michalski N., Goutman J.D., Auclair S.M., Boutet de Monvel J., Tertrais M., Emptoz A., Parrin A., Nouaille S., Guillon M., Sachse M. (2017). Otoferlin acts as a Ca^2+^ sensor for vesicle fusion and vesicle pool replenishment at auditory hair cell ribbon synapses. eLife.

[16] Roux I., Safieddine S., Nouvian R., Grati M., Simmler M.C., Bahloul A., Perfettini I., Le Gall M., Rostaing P., Hamard G. (2006). Otoferlin, defective in a human deafness form, is essential for exocytosis at the auditory ribbon synapse. Cell.

[17] Krahn M., Beroud C., Labelle V., Nguyen K., Bernard R., Bassez G., Figarella-Branger D., Fernandez C., Bouvenot J., Richard I. (2009). Analysis of the DYSF mutational spectrum in a large cohort of patients. Hum. Mutat..

[18] Kerr J.P., Ward C.W., Bloch R.J. (2014). Dysferlin at transverse tubules regulates Ca^2+^ homeostasis in skeletal muscle. Front. Physiol..

[19] (2018). Correction: European League Against Rheumatism/American College of Rheumatology classification criteria for adult and juvenile idiopathic inflammatory myopathies and their major subgroups. Ann. Rheum. Dis..

[20] Bohan A., Peter J.B. (1975). Polymyositis and dermatomyositis (first of two parts). N. Engl. J. Med..

[21] Bohan A., Peter J.B. (1975). Polymyositis and dermatomyositis (second of two parts). N. Engl. J. Med..

[22] Bode R.K., Klein-Gitelman M.S., Miller M.L., Lechman T.S., Pachman L.M. (2003). Disease activity score for children with juvenile dermatomyositis: Reliability and validity evidence. Arthritis Rheum..

[23] Tansley S.L., Simou S., Shaddick G., Betteridge Z.E., Almeida B., Gunawardena H., Thomson W., Beresford M.W., Midgley A., Muntoni F. (2017). Autoantibodies in juvenile-onset myositis: Their diagnostic value and associated clinical phenotype in a large UK cohort. J. Autoimmun..

[24] Cox A., Tolkach Y., Stein J., Kristiansen G., Ritter M., Ellinger J. (2021). Otoferlin is a prognostic biomarker in patients with clear cell renal cell carcinoma: A systematic expression analysis. Int. J. Urol..

[25] Bankhead P., Loughrey M.B., Fernandez J.A., Dombrowski Y., McArt D.G., Dunne P.D., McQuaid S., Gray R.T., Murray L.J., Coleman H.G. (2017). QuPath: Open source software for digital pathology image analysis. Sci. Rep..

[26] Zak M., Pfister M., Blin N. (2011). The otoferlin interactome in neurosensory hair cells: Significance for synaptic vesicle release and trans-Golgi network (Review). Int. J. Mol. Med..

[27] Manchanda A., Chatterjee P., Bonventre J.A., Haggard D.E., Kindt K.S., Tanguay R.L., Johnson C.P. (2019). Otoferlin Depletion Results in Abnormal Synaptic Ribbons and Altered Intracellular Calcium Levels in Zebrafish. Sci. Rep..

[28] Bansal D., Campbell K.P. (2004). Dysferlin and the plasma membrane repair in muscular dystrophy. Trends Cell Biol..

[29] Varga R., Kelley P.M., Keats B.J., Starr A., Leal S.M., Cohn E., Kimberling W.J. (2003). Non-syndromic recessive auditory neuropathy is the result of mutations in the otoferlin (OTOF) gene. J. Med. Genet..

[30] Khojah A., Liu V., Morgan G., Shore R.M., Pachman L.M. (2021). Changes in total body fat and body mass index among children with juvenile dermatomyositis treated with high-dose glucocorticoids. Pediatr. Rheumatol. Online J..

[31] Gibbs E.K.A., Morgan G., Ehwerhemuepha L., Pachman L.M. (2023). The von Willebrand Factor Antigen Reflects the Juvenile Dermatomyositis Disease Activity Score. Biomedicines.

[32] Kishi T., Chipman J., Evereklian M., Nghiem K., Stetler-Stevenson M., Rick M.E., Centola M., Miller F.W., Rider L.G. (2020). Endothelial Activation Markers as Disease Activity and Damage Measures in Juvenile Dermatomyositis. J. Rheumatol..

[33] Briones M.R., Morgan G.A., Amoruso M.C., Rahmani B., Ryan M.E., Pachman L.M. (2017). Decreased CD3-CD16+CD56+ natural killer cell counts in children with orbital myositis: A clue to disease activity. RMD Open.

[34] Costin C., Morgan G., Khojah A., Klein-Gitelman M., Pachman L.M. (2023). Lower NK Cell Numbers in Children with Untreated Juvenile Dermatomyositis During the COVID-19 Pandemic. Clin. Immunol. Commun..

[35] Khojah A., Liu V., Savani S.I., Morgan G., Shore R., Bellm J., Pachman L.M. (2022). Association of p155/140 Autoantibody With Loss of Nailfold Capillaries but not Generalized Lipodystrophy: A Study of Ninety-Six Children With Juvenile Dermatomyositis. Arthritis Care Res..

[36] Oddis C.V., Reed A.M., Aggarwal R., Rider L.G., Ascherman D.P., Levesque M.C., Barohn R.J., Feldman B.M., Harris-Love M.O., Koontz D.C. (2013). Rituximab in the treatment of refractory adult and juvenile dermatomyositis and adult polymyositis: A randomized, placebo-phase trial. Arthritis Rheum..

[37] Aggarwal R., Bandos A., Reed A.M., Ascherman D.P., Barohn R.J., Feldman B.M., Miller F.W., Rider L.G., Harris-Love M.O., Levesque M.C. (2014). Predictors of clinical improvement in rituximab-treated refractory adult and juvenile dermatomyositis and adult polymyositis. Arthritis Rheumatol..

[38] Ochfeld E., Hans V., Marin W., Ahsan N., Morgan G., Pachman L.M., Khojah A. (2022). Coding joint: Kappa-deleting recombination excision circle ratio and B cell activating factor level: Predicting juvenile dermatomyositis rituximab response, a proof-of-concept study. BMC Rheumatol..

[39] Aggarwal R., Charles-Schoeman C., Schessl J., Bata-Csorgo Z., Dimachkie M.M., Griger Z., Moiseev S., Oddis C., Schiopu E., Vencovsky J. (2022). Trial of Intravenous Immune Globulin in Dermatomyositis. N. Engl. J. Med..

[40] Goswami R.P., Haldar S.N., Chatterjee M., Vij P., van der Kooi A.J., Lim J., Raaphorst J., Bhadu D., Gelardi C., Danieli M.G. (2022). Efficacy and safety of intravenous and subcutaneous immunoglobulin therapy in idiopathic inflammatory myopathy: A systematic review and meta-analysis. Autoimmun. Rev..

[41] Piper C.J.M., Wilkinson M.G.L., Deakin C.T., Otto G.W., Dowle S., Duurland C.L., Adams S., Marasco E., Rosser E.C., Radziszewska A. (2018). CD19^+^CD24^hi^CD38^hi^ B Cells Are Expanded in Juvenile Dermatomyositis and Exhibit a Pro-Inflammatory Phenotype After Activation Through Toll-Like Receptor 7 and Interferon-alpha. Front. Immunol..

[42] Domeier P.P., Chodisetti S.B., Schell S.L., Kawasawa Y.I., Fasnacht M.J., Soni C., Rahman Z.S.M. (2018). B-Cell-Intrinsic Type 1 Interferon Signaling Is Crucial for Loss of Tolerance and the Development of Autoreactive B Cells. Cell Rep..

[43] Stalmann U., Franke A.J., Al-Moyed H., Strenzke N., Reisinger E. (2021). Otoferlin Is Required for Proper Synapse Maturation and for Maintenance of Inner and Outer Hair Cells in Mouse Models for DFNB9. Front. Cell. Neurosci..

