# Supplementary material for: Increased Otoferlin Expression in B Cells Is Associated with Muscle Weakness in Untreated Juvenile Dermatomyositis: A Pilot Study
Source: Int J Mol Sci. 2023 Jun 23;24(13):10553. doi: 10.3390/ijms241310553 (PMC10341737; doi:10.3390/ijms241310553)
Supplement: Supplementary file 1 [file ijms-24-10553-s001.zip › ijms-2404852-supplementary.pdf]

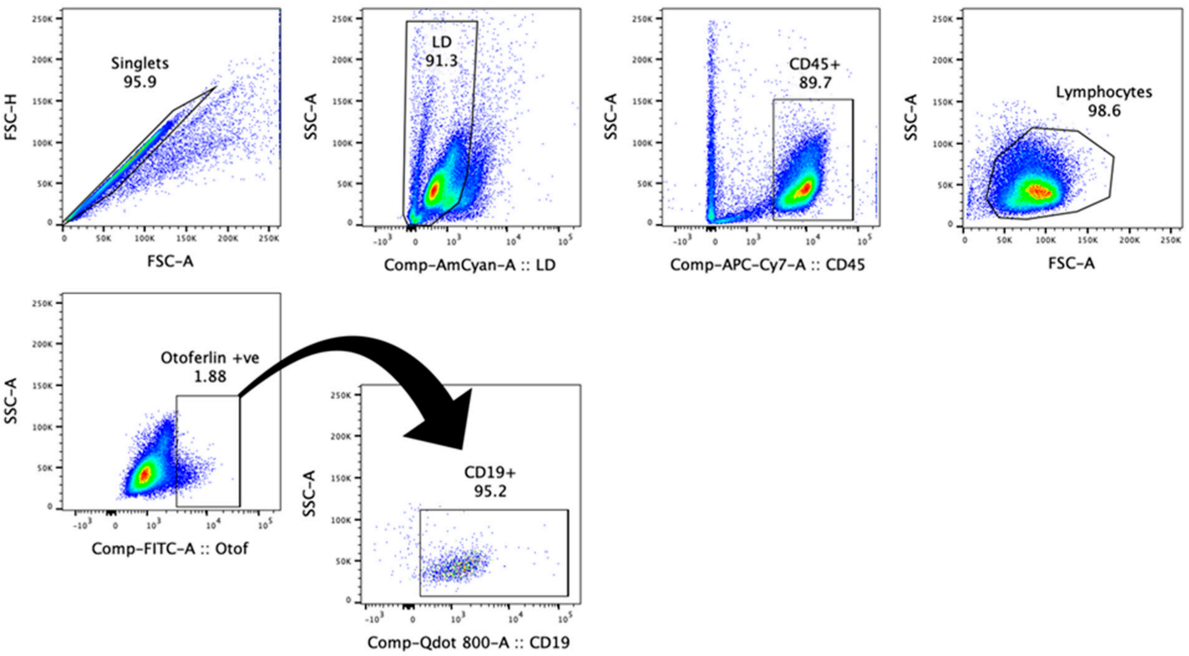

Figure S1. The gating strategy for otoferlin expression. The majority of the otoferlin-positive cells were B cells (63% - 99.4%).

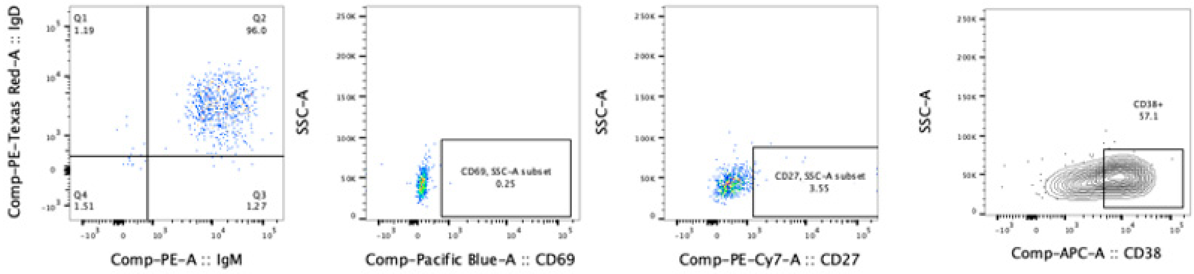

Figure S2. The gating strategy for otoferlin expression and B cell subset. Detailed B cell phenotyping showed that these B cells were IgD^{+}, IgM^{+} CD27^{-} naive B cells, with 65-75% of them expressing plasmablast markers (CD19^{+}, IgM^{+}, CD38^{hi}, CD24^{-}).

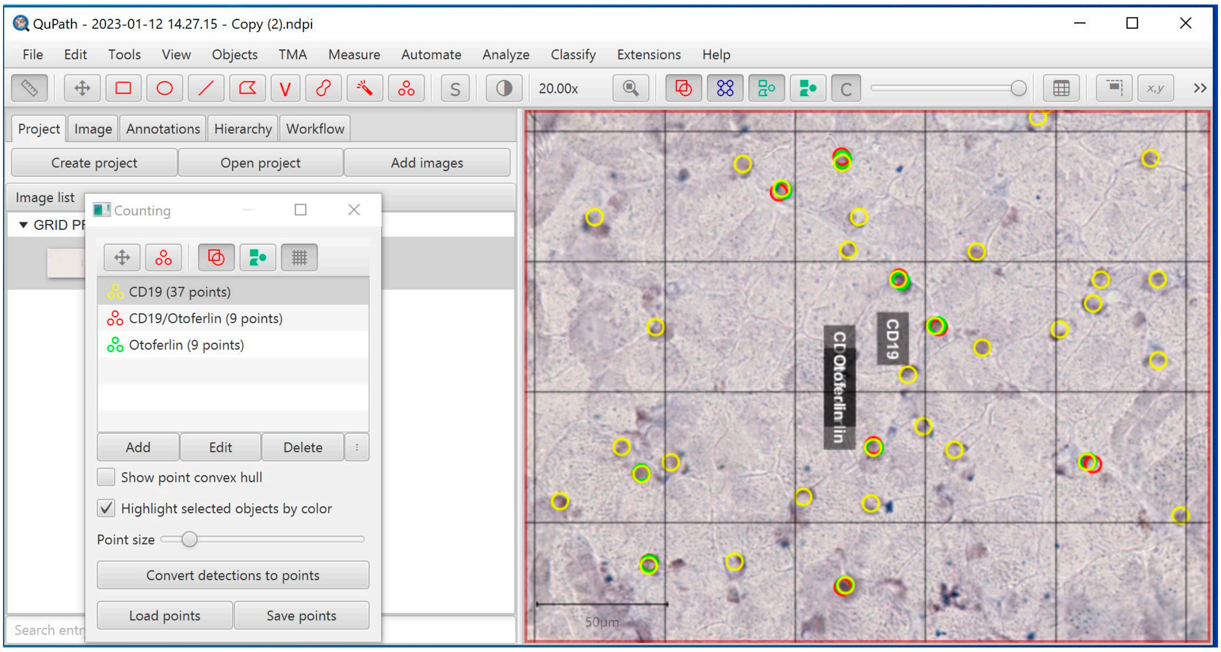

Figure S3. Print screen of QuPath (example of scoring CD19 and otoferlin staining). Scalebar 50µm.

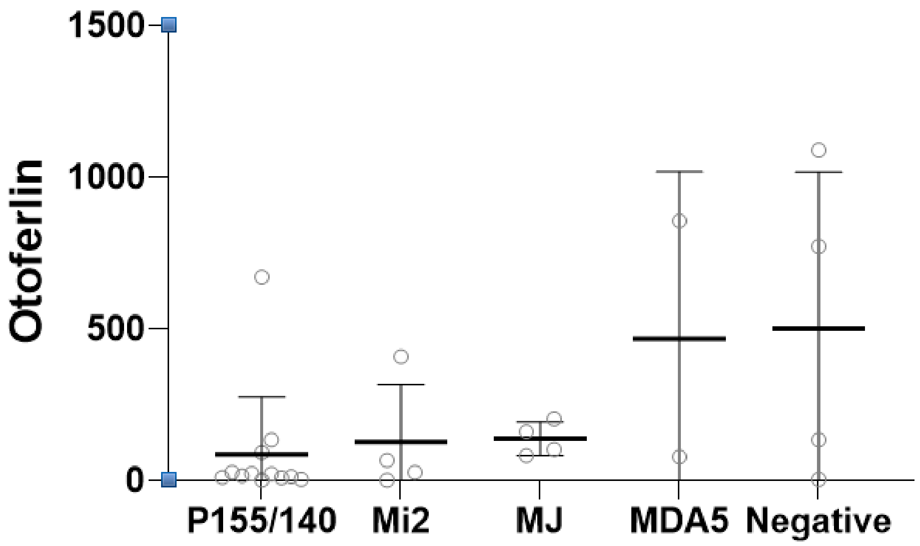

Figure S4. Otoferlin expression and MSAs. There was no significant association between the MSAs and otoferlin expression.

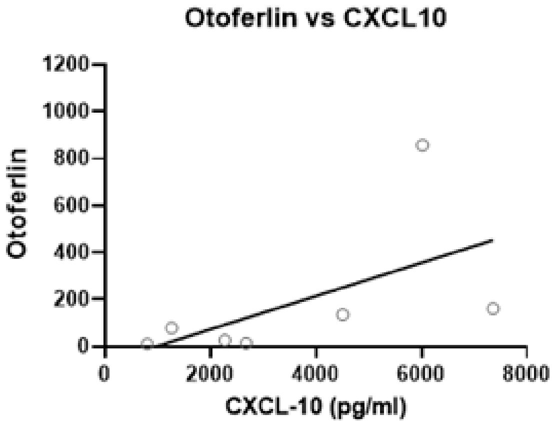

Figure S5. Correlation between otoferlin expression and serum CXCL10 (IP-10). There was a positive correlation between CXCL10 and otoferlin expression (Correlation Coefficient 0.8, p= 0.03)
